# Impact on health outcomes of hemodialysis patients based on the experience level of registered nurses in the hemodialysis department: a cross-sectional analysis

**DOI:** 10.3389/frhs.2023.1154989

**Published:** 2023-08-29

**Authors:** EunYoung Jeong

**Affiliations:** Department of Nursing, Wonkwang University, Iksan, Republic of Korea

**Keywords:** registered nurses, hemodialysis adequacy, blood pressure, serum albumin, hemoglobin, renal dialysis

## Abstract

**Background:**

The aim of the study is to improve the policy of health authorities regarding registered nurses (RNs) staffing by understanding how the health outcomes of hemodialysis (HD) patients vary depending on the level of HD nursing experience of at least 2 years.

**Methods:**

The study included 21,839 patients who started maintenance HD for 3 months in early 2013 in the same medical institutions. Demographic variables such as sex, age, and causes of HD; institutional variables such as type of institution and number of RNs; and health outcomes such as HD adequacy, systolic and diastolic blood pressure (SBP, DBP), hemoglobin (Hb), and serum albumin were collected through web-based questionnaires. To determine the relationship between variables, *t*-test, chi-square test, and ANOVA were employed. Binary logistic regression was used to examine the odds ratio.

**Results:**

Institutions with 100% of experienced RNs with at least 2 years of experience in HD units were found to have higher NKF K-DOQI criteria satisfaction rate than Institutions with <50% of experienced RNs with at least 2 years of experience in HD units for all health outcomes, except DBP (42.9% vs. 38.8%, *p* < .001) and Hb (27.8% vs. 24.4%, *p* < .001). Four of the six health-related variables—HD adequacy (Kt/v, urea reduction rate, URR), SBP, and serum albumin—were higher in institutions with 100% of experienced RNs than those with less than 50%.

**Conclusions:**

In order to improve HD patients' health outcomes, HD institutions should prioritize recruiting RNs who are proficient in HD care. A higher proportion of skilled RNs results in a higher ability to prepare for emergencies and early detection of patient complications. RNs with extensive experience in HD nursing, therefore, promote quality management of HD patients.

## Introduction

End-stage renal disease (ESRD) is a serious chronic disease affecting populations worldwide, and the number of cases is increasing sharply each year. Along with having the seventh highest global prevalence of cases, South Korea has seen a greater than 13-fold increase in the number of cases within a 27-year period—from 7,307 in 1990 to 98,746 in 2017. Among renal replacement therapy (RRT) patients, hemodialysis (HD) patients accounted for approximately 57% up to 2002, which increased sharply to 74.0% in 2017, indicating that HD has become a universally accepted RRT method (1). Diabetes mellitus (DM), hypertension (HT), and chronic glomerulonephritis (CGN) are the three major causative diseases of ESRD. Consequently, it is expected that as the number of diabetic and hypertensive patients increases with population aging, ESRD, which is a complication of these diseases, will also continue to increase.

HD is a form of RRT that alleviates symptoms by periodically filtering waste products and fluids. ESRD is a typical chronic disease that causes physical, psychological, and economic suffering as patients become dependent on HD machines until kidney transplantation or death, and are required to receive 2–3 sessions of HD therapy per week (2). Moreover, it is also a complex chronic disease that essentially requires active self-care, such as strict fluid restriction, diet, medication, and vascular access management, to minimize discomfort or complications associated with decreased renal function (3, 4). HD unit RNs are responsible for providing comprehensive care, including psychological support and education about diet and medication, as well as setting the HD machine, checking for arteriovenous fistula infections, and overseeing the entire HD process in emergency situations (5–8). Therefore, securing HD unit RNs with adequate experience has a profound impact on the health outcomes of HD patients. Given the diverse and specialized nature of the work, the American Nurses Association (ANA) has been operating the nephrology nursing certification program since 2005. Meanwhile, Medicare stipulates that essential workers for operating HD units must include doctors, nurses, nutritionists, and social workers, and each HD institution must have one nurse manager with at least 12 months of general nursing experience and at least 6 months of dialysis experience on site (9). This recognizes the specialization of HD unit RNs and assumes that health outcomes of patients could be changed by providing high-quality nursing care.

Studies in South Korea have consistently shown that higher RN staffing levels improve the health outcomes of patients in intensive care units (ICUs) or general wards (10–16). However, to the best of my knowledge, there have been no studies in South Korea examining, not only the number of HD unit RNs, but also HD nursing experience; and only a few such studies outside South Korea. According to these aforementioned, having an adequate number of HD RNs can help reduce hepatitis C morbidity and seroconversion rates (17, 18). Moreover, inadequate HD nurse staffing not only interferes with proper delivery of care services to the patients, but also increases patient complaints, shortens HD time (19), and increases hospitalization rates (20). These results indicate that HD RNs staff levels impact the health outcomes of HD patients.

The objective of this study is to improve health authorities' policies regarding RNs staffing by identifying how the health outcomes of HD patients vary depending on the level of HD RNs nursing experience. This leads us to the following hypothesis:

The more RNs who have worked in the HD room for more than 2 years, the more likely the health outcomes of HD subjects will meet the National Kidney Foundation Disease Outcomes Quality Initiative (NKF K-DOQI) guidelines.

## Materials and methods

### Materials

This study, conducted in 2016, used data from the 2013 Hemodialysis Adequacy Assessment (HAA) published by the Health Insurance Review and Assessment Service (HIRA). After this study was conducted, the new data became harder to obtain due to institutional privacy, despite being secondary data without personal identification. Therefore, the data used in this study are highly relevant.

The data consist of three parts: health insurance claims data, care facility status data, and HD patient survey. The survey was registered by hospital staff on the HIRA electronic system according to the patient's medical records.

### Study population

The subjects of HIRA HAA were adults aged ≥18 years who received outpatient HD [Korean Standard Classification of Diseases (KCD) O7020 and O9991] regularly at least twice a week for 3 months at the same HD institution. As of the first half of 2013, 69,837 patients received maintenance HD at the same institution. The subjects were randomly sampled from this population based on stratified sampling by sex and age. For institutions with ≤40 qualified patients, all patients were surveyed, while for institutions with >40 qualified patients, up to 40 patients were included. Consequently, 21,839 patients were selected from 728 institutions.

### HD unit RNs

#### HD unit RNs

HD unit RNs refer to all RNs assigned exclusively to the HD unit. RNs who also worked in other departments (ICUs, operating room, etc.), RNs who worked less than 30 days during the 3-month survey period, and nursing assistants, including assistant nurses (ANs), were excluded.

#### RNs with at least two years of work experience in HD units

This variable was defined as RNs with at least 2 years of HD nursing experience, including work experience in HD units in the current, as well as previous medical institutions, as of November 15, 2013. The percentage of “RNs with at least two years of work experience in HD units” was calculated by dividing the sum of the total work days of RNs with at least 2 years of work experience in HD units by the sum of the total work days of all RNs working in the HD unit (Table 1). Based on this proportion, the HD institutions were divided into four groups according to the proportion of RNs with HD experience of 2 years or more: ≤50% (Group I), >50% but ≤75% (Group II), >75% but <100% (Group III), and 100% (Group IV).

**Table 1:** Data description in the analysis and operational definition.

Variables			Operational definition	Unit
Dependent variables	Attainment of HD adequacy		$\frac{The\;No\;of\;patients\;attaining\;Kt\;\text{.}\;\text{.}\;\text{.}\;V\;\geq \;1.2\;\;or\;URR\;\geq \;65\text{\%}}{The\;No\;of\;patients\;taking\;HD\;adequacy\;test}\;\times 100$	URR: %
	Attainment of SBP		$\frac{The\;No\;of\;patients\;attaining\;SBP\;<\;140mmHg}{The\;No\;of\;patients\;taking\;SBP}\times 100$	mmHg
	Attainment of DBP		$\frac{The\;No\;of\;patients\;attaining\;DBP\;<\;90mmHg}{The\;No\;of\;patients\;taking\;DBP}\;\times 100$	mmHg
	Attainment of Serum Alb		$\frac{The\;No\;of\;patients\;attaining\;Serum\;Alb\;\geq \;4.0g/dl}{The\;No\;of\;patients\;taking\;Serum\;Alb\;test}\;\times 100$	g/dl
	Attainment of Hb		$\frac{The\;No\;of\;patients\;attaining\;Hb\;\geq \;11g/dl}{The\;No\;of\;patients\;taking\;Hb\;test}\;\times 100$	g/dl
Independent variables	Client	Age		
		Sex	Male; 0, Female; 1	
		Causes of HD	1; HT, 2; DM, 3; CGN, 4; Others 5; Unknown	
		Comorbidity	1: Heart disease, 2: Cerebrovascular disease, 3: Liver cirrhosis, 4: Gastrointestinal bleeding, 5: Chronic lung disease, 6: Malignant, 7: Diabetes Mellitus	
		Year of HD Maintenance	From the first day of HD To 2013.11.15.	year
	Institutional	Type of Institute	1: Tertiary Hospital, 2: General Hospital, 3: Hospital, 4: Clinic	
		No of Doctors	Total No of doctors in HD department	No
		Presence of Nephrologist	Whether presence or absence of Nephrologist	
		% Of above 2 Years’ experience HD RN	$\frac{Sum\;of\;workingdays\;of\;above\;2\;years\;experience\;RNs}{Sum\;of\;working\;days\;of\;RNs}\;\times 100$	%
		Average No of HD per RN per Day	$\frac{Total\;No\;of\;HD}{Sum\;of\;working\;days\;of\;RNs}\;\times 100$	No

HD, hemodialysis; URR, urea reduction rate; SBP, systolic blood pressure; DBP, diastolic blood pressure; Alb, albumin; Hb, hemoglobin; HT, hypertension; DM, diabetes mellitus; CGN, chronic glomerulonephritis; RN, registered nurse.

### Health outcome variables

The NKF K-DOQI guidelines were used to determine health outcomes with respect to the management of HD patients. These guidelines use four major clinical outcomes as major health indicators for HD patients: (1) HD adequacy (Kt/V or URR); (2) pre-dialysis systolic blood pressure (SBP) and diastolic blood pressure (DBP); (3) serum albumin; and (4) Hb or hematocrit (Hct). The recommended levels for each are Kt/V ≥ 1.2 (or URR ≥ 65%) (21), pre-dialysis SBP < 140 mmHg and pre-dialysis DBP < 90 mmHg (22); Hb ≥ 11 g/dl (or Hct ≥ 33%) (23), and serum albumin ≥ 4.0 g/dl (24) (Table 1).

### Data analysis

In the first stage of data analysis, demographic characteristics and health variables, including causative disease, were compared using descriptive statistical analysis. For statistical analysis, the *t*-test was used for continuous variables; chi-square test for nominal variables; and ANOVA for three or more factors. All results were reported with *p*-values. In the second stage, satisfaction of NKF K-DOQI criteria according to the percentage of RNs with at least 2 years of work experience in HD units was analyzed. Lastly, the influence on health outcomes according to the percentage of RNs with at least 2 years of work experience exclusively in HD units was analyzed using binary logistic regression analysis, which is a statistical method that is useful when dealing with categorical observed data for dependent variables. If six dependent variables (HD adequacy; Kt/v URR, SBP, DBP, Serum albumin, Hb, Hct) met the K-DOQI guidelines, it was set to 1, and if not, it was set to 0. Factors affecting dependent variables were divided into individual level and institutional level. the individual level variables were age, sex, year of HD maintenance, primary cause of ESRD etc. and their institutional characteristics were type of institutes, number of doctors and nephrologist etc. considered (Table 1). The predictors were analyzed when both patients and their institutional characteristics were considered during the analysis. For binary logistic analysis, the method of inputting all variables into the model was used as the method for selecting the optimal variable. Moreover, the likelihood of satisfying each health outcome (dependent variable) when each independent variable increases by 1, relative to institutions with <50% of RNs with at least 2 years of work experience in HD units, was expressed in terms of odds ratio (OR) and 95% confidence interval (CI).

## Results

### General characteristics

A total of 21,839 HD patients were included in the study (males, 57.9%; females, 42.1%). The mean age was 59.1 years and females were significantly older than males. The mean duration of HD therapy was 13.3 years and there was no difference between males and females. The mean values of health outcome indicators were as follows: HD adequacy, Kt/V 1.51 ± 0.27, and URR 70.7 ± 6.02%; SBP 141.2 ± 15.89 mmHg and DBP 78.7 ± 9.38 mmHg; serum albumin 3.99 ± 0.34 g/dl; and Hb 10.6 ± 0.86 g/dl. Males showed poorer health outcomes than females for all indicators (*p* < .001), except for albumin (4.02 g/dl vs. 3.95 g/dl, *p* < .001) and Hb (10.7 g/dl vs. 10.5 g/dl, *p* < .001) (Table 2).

**Table 2:** Characteristics of hemodialysis patients.

	Total, *n* (%)	Male	Female	*p*-value
No. of Patients	21,839 (100.0)	12,766 (57.9)	9,073 (42.1)	
Age	59.1 ± 12.96	58.5 ± 12.87	59.8 ± 13.05	<0.001
Year of HD maintenance	13.3 ± 0.66	13.2 ± 0.35	13.4 ± 0.86	.635
Primary causes of ESRD				
DM	8,831 (40.4)	5,327 (41.7)	3,504 (38.6)	<0.001
HT	5,906 (27.0)	3,518 (27.6)	2,388 (26.3)	<0.001
CGN	2,603 (11.9)	1,452 (11.4)	1,151 (12.7)	<0.001
Others	1,859 (8.5)	1,003 (7.9)	856 (9.4)	<0.001
Unknown	2,640 (12.1)	1,466 (11.5)	1,174 (12.9)	<0.001
Co-morbidity				
DM	9,479 (43.4)	5,729 (60.4)	3,750 (39.6)	<0.001
Heart disease	4,181 (19.1)	2,544 (60.8)	1,637 (39.2)	<0.001
CVD	1,235 (5.7)	791 (64.0)	444 (36.0)	<0.001
Malignant	692 (3.2)	429 (62.0)	263 (38.0)	.030
GI Bleeding	647 (3.0)	377 (58.3)	270 (41.7)	.477
LC	489 (2.2)	364 (74.4)	125 (25.6)	<0.001
CLD	325 (1.5)	226 (69.5)	99 (30.5)	<0.001
Health outcomes (mean)				
Kt/V (*n* = 18,117)	1.51 ± 0.27	1.40 ± 0.21	1.66 ± 0.26	<0.001
URR, % (*n* = 20,822)	70.7 ± 6.02	68.3 ± 5.25	74.2 ± 5.28	<0.001
SBP, mmHg (*n* = 21,839)	141.2 ± 15.89	142.3 ± 15.05	139.5 ± 16.85	<0.001
DBP, mmHg (*n* = 21,839)	78.7 ± 9.38	79.5 ± 9.19	77.6 ± 9.53	<0.001
Albumin, g/dl (*n* = 21,837)	3.99 ± 0.34	4.02 ± 0.35	3.95 ± 0.32	<0.001
Hb, g/dl (*n* = 21,838)	10.6 ± 0.86	10.7 ± 0.92	10.5 ± 0.75	<0.001

HD, hemodialysis; ESRD, end-stage renal disease; DM, diabetes mellitus; HT, hypertension; CGN, chronic glomerulonephritis; CVD, cerebrovascular disease; GI, gastrointestinal; LC, liver cirrhosis; CLD, chronic lung disease; URR, urea reduction rate; SBP, systolic blood pressure; DBP, diastolic blood pressure; Hb, hemoglobin.

### HD unit RNs

The total number of HD institutions was 728, with more than half (56.7%) being clinics, and 53.4% of HD patients were receiving HD from clinic-level institutions. The total number of HD unit RNs was 5,622 (7.73 ± 5.05/institution). The results showed that each RN performed HD 4.35 ± 1.77 times per day, with RNs in tertiary hospital HD units performing the fewest (3.8 ± 0.61) and RNs in hospital-level institutions performing the most (5.09 ± 2.42). The number of RNs with at least 2 years of experience in HD units among all HD unit RNs was 4,203 (74.8%), representing an average of 5.78 ± 4.00 per institution. Tertiary hospitals had the highest average number of RNs with at least 2 years of experience in HD units (12.7 ± 6.05), while hospital-level institutions had the lowest (3.45 ± 2.08) (Table 3).

**Table 3:** Characteristics of hemodialysis institutions.

		Total, *n* (%)	Tertiary hospital	General hospital	Hospital	Clinic
No of Institutions		728 (100.0)	42 (5.8)	199 (27.3)	74 (10.2)	413 (56.7)
No of Patients		21,839 (100.0)	1,586 (7.3)	6,011 (27.5)	2,580 (11.8)	11,662 (53.4)
No of Nurses	Total	5,622 (100.0)	677 (12.0)	1,529 (27.2)	364 (6.5)	3,052 (54.3)
	Average	7.73 ± 5.05	16.1 ± 7.70	7.69 ± 4.64	4.9 ± 2.62	7.39 ± 4.41
No of HD per RN per Day		4.35 ± 1.77	3.80 ± 0.61	4.50 ± 1.70	5.09 ± 2.42	4.21 ± 1.70
No of HD Nurses Above 2 years	Total	4,203 (74.8)	535 (12.7)	1,102 (26.2)	255 (6.1)	2,311 (55.0)
	Average	5.78 ± 4.00	12.7 ± 6.04	5.54 ± 3.50	3.45 ± 2.08	5.6 ± 3.45

HD, hemodialysis; RN, registered nurse.

### RNs with at least two years of work experience in HD units

Table 3 shows that in 80 institutions (11.0%), less than half of the HD unit RNs had at least 2 years of experience (Group I), while in 168 institutions (23.1%), all HD unit RNs had at least 2 years of experience (Group IV). Among the Group IV institutions, 108 institutions (64%) were clinics, while among Group I institutions, 36 institutions (45%) were clinics. Among the 42 tertiary hospitals, 64.3% belonged to Group III (>75% but <100% of HD unit RNs with at least 2 years of experience), while most of the clinics, hospitals, and general hospitals belonged to Group II (>50% but ≤100% of HD unit RNs with at least 2 years of experience (Table 4).

**Table 4:** The number of RNs with more than 2 years of experience.

	Total (*n*, %)	I (below 50%)	II (50%–75%)	III (75%–100%)	IV (100%)
No of Institutes	728 (100.0)	80 (11.0)	241 (33.1)	239 (32.8)	168 (23.1)
Tertiary hospital	42 (5.8)	0 (0.0)	13 (31.0)	27 (64.3)	2 (4.8)
General hospital	199 (27.3)	29 (14.6)	68 (34.2)	66 (33.2)	36 (18.1)
Hospital	74 (10.2)	15 (20.3)	26 (35.1)	11 (14.9)	22 (29.7)
Clinic	413 (56.7)	36 (8.7)	134 (32.4)	135 (32.7)	108 (26.2)
No of Patients	21,839 (100.0)	2,509 (11.5)	7,281 (33.3)	8,173 (37.4)	3,876 (17.7)

RN, registered nurse.

### Criteria satisfaction rate according to percentage of RNs with at least two years of work experience in HD units

With respect to the NKF K-DOQI criteria satisfaction rate according to experienced RNs, there were statistically significant differences in all health outcomes between the groups, except serum albumin (*p* = .205). Group I showed the lowest satisfaction rate for HD adequacy (Kt/V, URR), SBP, and serum albumin. Group IV showed higher NKF K-DOQI criteria satisfaction rate than Group I for all health outcomes, except DBP (42.9% vs. 38.8%, *p* < .001) and Hb (27.8% vs. 24.4%, *p* < .001) (Table 5).

**Table 5:** Attainment of standards by the percentage of RNs with more than 2 years of experience.

	%, (*n*)	I (below 50%)	II (50%–75%)	III (75%–100%)	IV (100%)	*p*-value
Kt/V (≥1.2) *n *= 18,117	91.3 (16,536)	85.1 (1,708)	91.6 (5,497)	92.7 (6,516)	91.5 (2,815)	<0.001
URR (≥65%) *n *= 20,822	85.3 (17,752)	81.6 (2,046)	84.9 (5,891)	87.2 (6,720)	84.3 (3,095)	<0.001
SBP (<140 mmHg) *n *= 21,839	18.1 (3,959)	16.4 (412)	18.9 (1,374)	18.5 (1,514)	17.0 (659)	.008
DBP (<90 mmHg) *n *= 21,839	43.8 (9,575)	42.9 (1,077)	43.2 (3,143)	47.1 (3,850)	38.8 (1,505)	<0.001
Albumin (≥4.0 g/dl) *n *= 21,837	52.0 (11,356)	50.9 (1,276)	52.0 (3,787)	51.7 (4,225)	53.4 (2,068)	.205
Hb (≥11.0 g/dl) *n *= 21,838	27.0 (5,897)	27.8 (698)	26.6 (1,936)	28.4 (2,318)	24.4 (945)	<0.001

RN, registered nurse; URR, urea reduction rate; SBP, systolic blood pressure; DBP, diastolic blood pressure; Hb, hemoglobin.

### Predictors of NKF K-DOQI criteria satisfaction by health outcome

Binary logistic regression analysis was performed to determine whether the percentage of RNs with at least 2 years of experience influenced health outcomes. Having more experienced RNs significantly increased the OR of satisfying the NKF K-DOQI criteria for Kt/V and URR among the six health outcome indicators (Kt/V: OR 1.011, 95% CI 1.008–1.013, *p* < .001; URR: OR 1.005, 95% CI 1.003–1.007, *p* < .001). Having more experienced RNs also increased the OR of satisfying the NKF K-DOQI criteria for SBP, DBP, and Hb, but the results were not statistically significant (*p*-values: DBP: 0.852, SBP: 0.350, Hb: 0.996). Serum albumin showed an inverse relationship that decreased as the number of experienced RNs increased, but there was no statistical significance (Table 6).

**Table 6:** Attainment of standards by percentage of RNs with more than 2 years of experience.

	*p*-value	OR	95% CI
Kt/V (≥1.2) *n *= 18,117	<0.001	1.011	1.008–1.013
URR (≥65%) *n *= 20,822	<0.001	1.005	1.003–1.007
SBP (<140 mmHg) *n *= 21,839	.350	1.001	.999–1.003
DBP (<90 mmHg) *n *= 21,839	.852	1.000	.999–1.002
Albumin (≥4.0 g/dl) *n *= 21,837	.081	.999	.997–1.000
Hb (≥11.0 g/dl) *n *= 21,838	.996	1.000	.998–1.002

RN, registered nurse; OR, odds ratio; CI, confidence interval; URR, urea reduction rate; SBP, systolic blood pressure; DBP, diastolic blood pressure; Hb, hemoglobin.

## Discussion

This study analyzed data on 21,839 patients receiving maintenance HD to investigate whether the percentage of RNs with at least 2 years of experience in HD units affects the health outcome variables of patients. The study population in this study included more males than females (59.7% vs. 42.1%) which was similar to the US (57.8% vs. 42.2%) (25) and Japan (62.3% vs. 37.7%) (26). The mean age of the patients was 59.1 years, which lower than that of the US (59.1 years) and Japan (66.9 years) (24, 25), but the average age of HD patients in South Korea is expected to increase gradually with population aging. The most common causative disease of HD was DM and the patients had been receiving HD for an average of 13.3 years, as of November 2013. DM was also found to be the most common comorbidity (43.4%). In 2016, South Korea had the fourth highest incidence of HD due to DM in the world, behind Malaysia, Singapore, and Mexico (27), which indicated the criticality of reducing the progression through education about DM and strict instructions about self-care.

There were 5,622 HD unit RNs representing 7.73 RNs per institution. Compared to a 2009 study in the US that found 20,709 RNs working in 4,800 HD institutions at an average of 4.31 RNs per institution (28), HD institutions in South Korea have a higher number of RNs working per institution than the US. In the US, the duties of HD RNs and technicians are not clearly defined, and HD technicians are also allowed to perform arteriovenous fistula care, physical examinations, and drug administration, similar to nurses (28). Therefore, it is suspected that the situation is different from South Korea as HD technicians may have been hired instead of RNs in the US.

RNs with at least 2 years of work experience in the HD unit accounted for 74.8% of all HD unit RNs. Hospital-level institutions, as opposed to clinic-level institutions, had a lower number of HD unit RNs per institution (4.9% vs. 7.4%) and experienced RNs (3.5% vs. 5.6%). It is suspected that such results are due to clinics being specialized to provide services to HD patients, whereas hospital-level institutions mainly deal with various types of patients other than HD patients.

The percentage of experienced HD RNs could be viewed as an indicator of the qualitative aspect of nursing care service provided. Unlike less experienced RNs, experienced RNs have relatively higher competency to prepare for emergencies and detect complications early. According to Benner, experience includes not only time at the nursing field, but also self-reflection that leads to the identification or refinement of one's preconceptions in the real circumstances. In addition, Benner said that working at the same or similar nursing field for a long time can improve nursing competency, but this is not the same as growing as an expert (29). Nevertheless, it was found that experienced nurses not only performed much more complex functions than those who did not, but also responded well to situations requiring accurate judgment (30, 31). This study also shows that hospitals with RNs with at least 2 years of work experience in the HD unit generally have better patient health results than hospitals that do not, showing that experience is not just accumulation of time. While tertiary hospitals had the lowest percentage of experienced RNs (67.7%), clinic-level institutions had the highest (75.9%). This may be due to RNs who move or resign from tertiary hospitals, finding it relatively easier to enter clinic-level institutions, rather than hospital-level or higher institutions. Institutions with 100% experienced RNs showed higher mean values for HD adequacy Kt/V, SBP, and serum albumin. Moreover, with respect to the NRF K-DOQI criteria satisfaction rate, the rate for four out of six health-related variables (HD adequacy Kt/V, URR, SBP, and serum albumin) was higher in institutions with 100% experienced RNs than in those with <50%. Specifically, having a higher percentage of experienced RNs increased the OR of satisfying the NRF K-DOQI criteria for HD adequacy (Kt/V, URR), SBP, DBP, and Hb; however, serum albumin showed a reverse directionality of increased OR of satisfying the NRF K-DOQI criteria with fewer experienced RNs. However, the results were not statistically significant and the results in this study may have been different if the adjustment for albumin infusion was considered.

The limitations of this study are as follows: First, the data used in the study were collected from eligible medical institutions inputting data in a self-report format in accordance with the HIRA HAA plan. Therefore, the possibility of “reporting errors” cannot be dismissed. Reporting errors were minimized by comparing input data from randomly selected institutions and actual patient records.

Second, this study used a cross-sectional study design with simultaneous collection of data regarding experienced RN status and various health outcomes during a specific period from October to December 2013. Therefore, there are limitations in identifying temporal relationships when examining causal relationships between health outcome variables and independent variables. Therefore, to analyze the establishment of an accurate causal relationship for whether changes in health outcome variables are caused by nurse staffing level, studies with stronger evidence based on longitudinal linked data are required. Third, there are no existing study results that could be used as a reference for whether the health outcome variables in this study can sensitively reflect experienced HD RNs staffing. Studies on HD unit RNs have analyzed hepatitis C morbidity as a health outcome of patients with regard to the number of HD unit RNs (14, 15). Moreover, in studies on general wards and ICUs, outcome variables such as falls, bed sores, patient satisfaction, various complications, failure-to-resuscitate rate, re-hospitalization rate, and mortality rate were selected (3, 4, 9, 10–13). However, no studies have analyzed the satisfaction rate of clinical outcomes as outcome variables, as was done in this study. This study attempted to analyze health outcomes in patients in relation to the high or low number of experienced RNs in a special nursing unit in the form of an HD unit. HD patients have various comorbidities, and the number of older patients is increasing given the rapidly aging population and longer life expectancy. Therefore, clinical indicators that reflect continued monitoring, education, consultation, and observation were selected as health outcome variables instead of mortality, which is the ultimate outcome indicator. The findings in this study showed that two out of six variables were significantly influenced by the experienced RNs staffing level. This may be because the outcome indicators used in the study were clinical test indicators that are more closely associated with the behaviors of doctors than RNs; however, the results may have been influenced by not considering the severity factors, including the use of iron preparations and anti-hypertensive drugs and/or blood transfusion status.

Therefore, the findings suggest the need for in-depth long-term studies in the future to analyze not only various clinical test results that represent intermediate outcome indicators, but also the ultimate outcome variables, such as complications and mortality rate.

## Conclusion

Patients start on HD, usually due to DM or HT; given the rapid increase in the prevalence of HD and population aging, they generally progress to long-term healthcare users, as compared to patients with other diseases. Therefore, proper quality management of HD patients, as they grow older, could not only prevent complications and deterioration of patient conditions, but also minimize increases in healthcare costs. Adequate RNs staffing is essential for quality management of HD patients, and while it is important to secure RNs with sufficient experience, it is also important to have sufficient RNs staff to provide quality service. Therefore, it is important to first have adequate RNs staffing to reduce the workload of HD unit RNs to use as a basis for increasing the number of experienced RNs. Moreover, to overcome the limitations of cross-sectional analysis, HAA data from the HIRA should not be used as one-time data for adequacy assessment, but longitudinal linked data should be established to attempt a comprehensive analysis of the importance of RNs staffing as an influencing factor for health risks and causes of mortality among HD patients, and to use the findings as evidence for establishing health policies on RNs staffing.

## Data Availability

The original contributions presented in the study are included in the article, further inquiries can be directed to the corresponding author.
